# Evolving targets for lipid-modifying therapy

**DOI:** 10.15252/emmm.201404000

**Published:** 2014-08-29

**Authors:** Rose Q Do, Stephen J Nicholls, Gregory G Schwartz

**Affiliations:** 1 VA Medical Center, University of Colorado School of MedicineDenver, CO, USA; 2 South Australian Health and Medical Research Institute and University of AdelaideAdelaide, SA, Australia

**Keywords:** atherosclerosis, cholesterol, lipoproteins, triglycerides

## Abstract

The pathogenesis and progression of atherosclerosis are integrally connected to the concentration and function of lipoproteins in various classes. This review examines existing and emerging approaches to modify low-density lipoprotein and lipoprotein (a), triglyceride-rich lipoproteins, and high-density lipoproteins, emphasizing approaches that have progressed to clinical evaluation. Targeting of nuclear receptors and phospholipases is also discussed.

See also Glossary for abbreviations used in this article

## Introduction

Ischemic heart disease and cerebrovascular disease due to atherosclerosis remain leading causes of death in the world. Lipoprotein abnormalities play a key role in the pathogenesis of these diseases. Low-density lipoprotein (LDL), triglyceride-rich lipoproteins, and high-density lipoprotein (HDL) may contribute to the development and progression of atherosclerosis and its complications. Numerous strategies to modify each of the principal classes of lipoproteins have been or are currently under investigation. This review primarily focuses on those approaches that have progressed to clinical evaluation or implementation.

**Figure 1:**
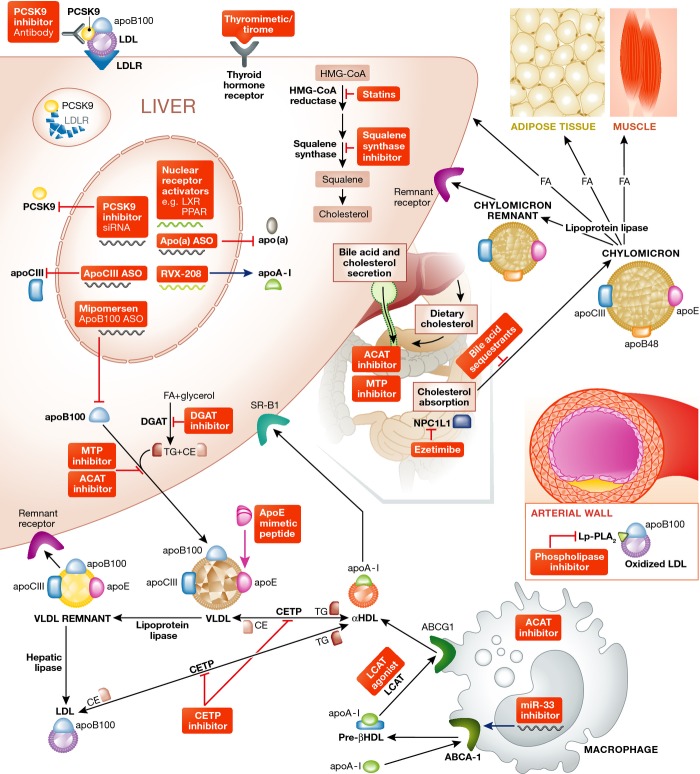
Schematic indicating potential targets for lipid metabolism

LDL, triglyceride-rich lipoproteins, and HDL comprise the three principal lipoprotein classes. The primary function of LDL is to deliver essential cholesterol to peripheral tissues. Triglyceride-rich lipoproteins carry a cargo of energy substrate (fatty acids) from intestine to liver and to peripheral tissues for fat storage and oxidative metabolism. HDL participates in reverse cholesterol transport to remove excess cholesterol stores from peripheral sites for biliary excretion. Each lipoprotein class may affect the development and progression of atherosclerosis and its complications. Numerous strategies to modify each of the principal classes of lipoproteins have been or are currently under investigation (Table1, Fig1).

**Table 1:** Strategies to reduce LDL and related atherogenic lipoproteins

Target	Agent(s)	Primary site of action	Principal effects on lipoproteins	Phase of clinical evaluation	Safety/tolerability issues
3-hydroxy-3-methylglutaryl coenzyme A (HMG CoA reductase)	Statins	Liver	LDL-C ↓↓ HDL-C →↑ TG ↓	Approved for the use in dyslipidemia and atherosclerosis	Muscle and liver enzyme abnormalities
Bile acid sequestrant	Cholestyramine and others	Intestine	LDL-C ↓ HDL-C ↑ TG ↑	Approved for the use in dyslipidemia	Gastrointestinal side effects; interference with absorption of other drugs; exacerbation of hypertriglyceridemia
Niemann-Pick C1-like protein	Ezetimibe	Intestine	LDL-C ↓	Approved for the use in dyslipidemia	Well tolerated, no outcomes data
Squalene synthase	Lapaquistat	Liver	LDL-C ↓ TG ↓	Development halted in Phase 2–3	Muscle and liver enzyme abnormalities
ApoB100	Mipomersen	Liver	apoB ↓ LDL-C ↓↓ TG ↓ Lp(a) ↓↓	Approved for the use in homozygous familial hypercholesterolemia	Hepatic steatosis, liver enzyme abnormalities, injection site reactions, flu-like symptoms
Acyl-CoA/cholesterol acyltransferase (ACAT)	Avasimibe, pactimibe, and others	Liver, intestine, macrophages	LDL-C ↓↓ TG ↓	Development halted in Phase 2	Neutral to adverse effect on atherosclerosis by imaging
Microsomal triglyceride transfer protein (MTP)	Lomitapide	Liver, intestine	LDL-C ↓↓ HDL-C ↓ TG ↓↓↓ Lp(a) ↓	Approved for use in homozygous familial hypercholesterolemia	Hepatic and intestinal steatosis, liver enzyme abnormalities
Thyroid hormone receptors	Thyromimetics (eprotirome, sobetirome, and others)	Liver	LDL-C ↓↓ TG ↓↓ Lp(a) ↓↓	Clinical development halted in Phase 3	Reversible reductions in thyroxine and thyroid binding globlulin, unclear clinical significance. Liver enzyme abnormalities
PCSK9	Alirocumab (REGN727/SAR236553), evolocumab (AMG 145), PF-04950615 (RN316), and others	Liver, intestine	LDL-C ↓↓↓ Lp(a) ↓ TG ↓ HDL-C ↑	Phase 3	Mild injection site reactions
Lipoprotein (a)	Niacin/nicotinic acid	Adipose, liver	Lp(a) ↓↓ LDL-C ↓ TG ↓↓ HDL-C ↑↑	Approved for the use in dyslipidemia	Flushing, liver enzyme and glucose abnormalities, hyperuricemia
	LDL apheresis	Serum	Lp(a) ↓↓↓ LDL ↓↓↓	Approved for the use in familial hypercholesterolemia	Nausea, vomiting, flushing, angina, syncope, bleeding
	Apo(a) antisense oligonucleotide 144367 ISIS apo(a) Rx	Liver	Apo(a) ↓↓↓ (preclinical)	Phase 1	
Strategies to reduce triglyceride-rich lipoproteins					
VLDL production/secretion, TG clearance	Eicosapentaenoic acid (EPA), docosahexaenoic acid (DHA)	Liver	TG ↓↓ LDL-C ↑	Approved for the use in dyslipidemia	Gastrointestinal side effects
ApoC-III	Antisense oligonucleotide ISIS 304801, ISIS apoC-III-Rx	Liver	ApoC-III ↓↓↓ TG ↓↓ HDL-C ↑	Phase 2	Injection site reaction
Diacylglycerol acyltransferase (DGAT)	LCQ-908, AZD7687, PF-04620110, and others	Intestine, liver, adipose	TG ↓↓	Phase 3	Gastrointestinal symptoms
ApoE	ApoE mimetic peptide (AEM-28)	Liver	Preclinical	Preclinical	
Strategies to increase HDL					
G protein-coupled receptor	Niacin/nicotinic acid	As above	As above	As above	As above
Cholesteryl ester transfer protein	Torcetrapib, dalcetrapib, evacetrapib, anacetrapib	Liver, circulation	HDL-C ↑↑↑ LDL-C ↓↓	Phase 3	Torcetrapib phase 3 trial stopped prematurely due to harm. Dalcetrapib phase 3 trial stopped due to futility.
Bromodomain and extra-terminal (BET) protein 2	RVX-208	Liver	HDL-C ↑ Large HDL↑	Phase 2	Liver enzyme abnormalities
Circulating lipoproteins	HDL-mimetic CER-001, ATI-5261 (preclinical), MDCO-216, and others	Serum	Preβ1 HDL ↑↑↑ TG ↑↑ Apo-AI ↑↑	Phase 2	Gastrointestinal symptoms, elevated triglycerides
ATP binding cassette transporter A1 and G1 (ABCA1 and ABCG1)	miR-33	Liver, endothelium	HDL-C ↑↑ (preclinical)	Preclinical	
Nuclear receptor agonists					
Liver X receptors	LXR-623 and others	Liver, intestine	HDL↑	Clinical development halted in Phase 2	Induced lipogenesis and hypertriglyceridemia. Dose dependent CNS effects
PPAR-α, γ, and/or δ	Fibrates (PPAR-α, TZDs (PPAR-γ, and novel PPAR agonists	PPAR-α: Liver, skeletal muscle PPAR-γ: adipose, vascular smooth muscle PPAR-δ: ubiquitous	PPAR-α: HDL-C ↑ TG ↓ PPAR-γ: HDL-C ↑ TG ↓ PPAR-δ: HDL-C ↑↑ LDL-C ↓ (α/δ) TG ↓ HDL-C ↑ LDL-C ↓	Fibrates and TZDs approved for clinical use in dyslipidemia and diabetes. Development of dual α/γ activators halted in Phase 2–3 Selective PPAR-δ and α/δ activators in early phase development	Decreased glomerular filtration rate (α), weight gain, fluid retention, congestive heart failure, bone fractures (γ). No long-term safety data for α/δ activators
Secretory and lipoprotein-associated phospholipase A2	Varespladib, darapladib	Multiple cell types	LDL-C ↓ VLDL ↓	Varespladib, darapladib terminated in Phase 3	Varespladib increased adverse events after acute coronary syndrome

Arrows indicate direction and magnitude of lipoprotein change.

↑ or ↓ indicates 0–30% change (increase/decrease).

↑↑ or ↓↓ indicates 30–60% change (increase/decrease).

↑↑↑ or ↓↓↓ indicates > 60% change (increase/decrease).

→ indicates neutral effect/no change.

## Targets for reduction of LDL and related atherogenic lipoproteins

Statin drugs have been the cornerstone of lipid-modifying therapy for more than a quarter century. Statins inhibit 3-hydroxy-3-methylglutaryl coenzyme A (HMG CoA) reductase, which is the rate-limiting step for cholesterol synthesis. A reduction in hepatic cholesterol synthesis and hepatocyte cholesterol concentration results in upregulation of LDL receptor expression on hepatocytes and enhanced clearance of LDL and other atherogenic lipoproteins from the circulation. Through this mechanism, statins can reduce concentrations of LDL-C by as much as 50–60%, with accompanying reductions of triglyceride-rich lipoproteins and modest increases in HDL-C. A large body of data from controlled clinical trials indicates that each 1 mmol/l reduction in LDL-C produced by statin treatment is associated with approximately 12% reduction in all-cause mortality and 20% reduction in cardiovascular morbidity (Baigent*et al*, 2005). At usual doses, statins reduce cardiovascular risk by 25–30%. Part of the clinical efficacy of statins may also be attributable to non-lipid, or ‘pleiotropic’ effects related to effects of HMG CoA reductase inhibition on isoprenoid intermediates in cholesterol biosynthesis (Davignon, 2004; Nohria*et al*, 2009; Zhou & Liao, 2010). However, substantial residual cardiovascular risk remains despite effective statin treatment. The extent to which this residual risk is attributable to lipoprotein abnormalities, and might be reduced by additional lipoprotein-modifying therapies, remains unknown. To date, no second lipid-modifying therapy, added to primary treatment with statins, has been proven to reduce cardiovascular risk. However, promising new targets and new approaches to established targets are being investigated.

### Cholesterol absorption

Interference with cholesterol absorption can lower circulating concentrations of LDL-C. Bile acid sequestrants such as cholestyramine lower LDL-C levels up to 25%. As monotherapy, cholestyramine was shown to reduce the risk of myocardial infarction (Lipid Research Clinics Program, 1984). However, use of bile acid sequestrants is limited by gastrointestinal side effects, interference with absorption of other drugs, and exacerbation of hypertriglyceridemia. A newer bile acid sequestrant, colesevelam, has a relatively low incidence of gastrointestinal side effects (Davidson*et al*, 1999).

A target for the inhibition of intestinal cholesterol absorption is Niemann-Pick C1-like 1 (NPC1L1). Ezetimibe inhibits cholesterol absorption by blocking the function of NPC1L1 (Garcia-Calvo*et al*, 2005) and lowers LDL-C in patients by approximately 15%. In a study of patients with heterozygous familial hypercholesterolemia followed for 24 months, there was no difference in intima-media thickness with ezetimibe and simvastatin, compared with placebo and simvastatin (Kastelein*et al*, 2008). However, there are no data to date indicating whether or not the additional reduction of LDL-C resulting from addition of ezetimibe to statin translates into additional reduction of cardiovascular risk. This hypothesis is being tested in the IMPROVE-IT trial, with results expected in 2014 (Califf*et al*, 2010).

### Squalene synthase

Squalene synthase acts downstream from HMG CoA reductase to convert farnesyl pyrophosphate to squalene, the first committed step in cholesterol biosynthesis. Inhibition of squalene synthase lowers plasma cholesterol levels without affecting the synthesis of upstream intermediates implicated in the development of myopathy with statins (Nishimoto*et al*, 2007). Lapaquistat acetate is a squalene synthase inhibitor that reduced LDL-C by 20% and lowered C-reactive protein levels by 25%. It progressed to evaluation in Phases 2 and 3 clinical trials. However, development of lapaquistat was halted due to two cases of severe liver enzyme elevation, coupled with evidence that the strategy might not reduce muscle toxicity (Stein*et al*, 2011). No other squalene synthase inhibitors have reached advanced stages of clinical development.

### Expression of apolipoprotein B100

Apolipoprotein (apo) B-containing lipoproteins include LDL, very low-density lipoprotein (VLDL), and VLDL remnants. Because these lipoproteins may promote atherosclerosis, strategies to prevent expression of apoB are attractive. However, interference with hepatic export of apoB-containing lipoproteins also has the potential to promote hepatic steatosis.

Mipomersen is a 20-base, 2′-O-(2-methoxy) ethyl-modified antisense oligonucleotide that targets mRNA encoding apoB100. It reduces circulating levels of all lipoprotein species containing apoB100 in humans. In Phases 2 and 3 clinical trials of mipomersen as monotherapy or added to statins in patients with familial hypercholesterolemia (FH), LDL-C reductions of up to 47% were observed (Visser*et al*, 2012). Because mipomersen does not significantly affect intestinal apoB48 expression, intestinal fat absorption is unaffected and intestinal steatosis is avoided (Crooke*et al*, 2005). However, treatment with mipomersen was associated with injection site reactions, flu-like symptoms, and hepatic steatosis with elevated liver transaminases (Visser*et al*, 2012).

Mipomersen is currently approved for the treatment of homozygous familial hypercholesterolemia. However, the long-term benefit/risk profile remains to be determined and might be influenced by tolerability or hepatic safety. Other approaches to knockdown apoB100, including small interfering RNAs, are under investigation (Tadin-Strapps*et al*, 2011).

### VLDL assembly – ACAT and MTP

Another approach to reduce LDL is to prevent hepatic secretion of its precursor lipoprotein, VLDL, by interfering with the lipidation of apoB. Two enzymes involved in this process, acyl-CoA/cholesterol acyltransferase (ACAT) and microsomal triglyceride transfer protein (MTP), have been targeted.

ACAT1 and ACAT2 are expressed in macrophages as well as liver and intestine where they are involved in accumulation of cholesterol ester, foam cells, atherosclerosis, and providing cholesterol ester to secreted VLDL and chylomicrons (Lee*et al*, 2000; Leon*et al*, 2005). Surprisingly, clinical trials that used intravascular ultrasound to evaluate non-specific ACAT inhibitors demonstrated that treatment had a neutral to adverse effect on coronary atherosclerosis (Tardif*et al*, 2004; Nissen*et al*, 2006). The explanation may be related to toxic effects of increased free cholesterol in macrophages. Selective ACAT2 inhibition might remain a plausible approach to retard atherosclerosis (Ohshiro & Tomoda, 2011).

MTP transfers neutral lipids to nascent apoB and thereby affects the rate of VLDL and chylomicron synthesis. Loss-of-function mutation of MTP is the cause of abetalipoproteinemia, a condition with defective fat absorption and the absence of circulating apoB-containing lipoproteins. Lomitapide is a small molecule inhibitor of MTP that was shown to lower LDL-C levels by 50% in patients with homozygous FH and has been approved for the use in that condition (Cuchel & Rader, 2013). Although lomitapide is effective in lowering LDL-C, its use is unlikely to extend beyond FH because of a high incidence of gastrointestinal symptoms related to malabsorption of fat and hepatic steatosis related to inhibition of hepatic lipid export (Cuchel*et al*, 2013).

### Thyromimetics or tiromes

Thyroid hormone exerts tissue-specific effects, with the thyroid hormone receptor β1 isoform (TR β1) predominating in liver and involved in cholesterol metabolism (Gullberg*et al*, 2000). TR β1 activation depletes intracellular cholesterol concentration, leading to increased expression of the LDL receptor in hepatocytes. Hypolipidemic effects of TR β1 activation are also increased biliary cholesterol excretion through stimulation of cholesterol 7a-hydroxylase (CYP7A1) expression (Lin*et al*, 2012), downregulation of sterol regulatory element-binding protein (SREBP)-1c (Hashimoto*et al*, 2006), and non-classical signaling pathways (Cordeiro*et al*, 2013).

The TR β1 agonists such as eprotirome have been evaluated in clinical trials. On a background of statin treatment, eprotirome lowered LDL-C up to 32%, associated with reductions of apoB, triglycerides, and Lp(a) (Ladenson*et al*, 2010). Eprotirome was effective in decreasing levels of atherogenic lipoproteins in patients with hypercholesterolemia (Angelin*et al*, 2014). However, a recent Phase 3 trial was terminated after liver injury was noted in humans and cartilage injury noted in preclinical data with dogs (Sjouke*et al*, 2014). Currently, there are no known plans to continue with its development.

### PCSK9

broprotein convertase subtilisin/kexin type 9 (PCSK9) is a secreted protein that regulates the hepatic LDL receptor (LDLR), and in turn circulating levels of LDL-C (Akram*et al*, 2010; Do*et al*, 2013). When an LDL particle binds to a hepatocyte LDLR in the absence of PCSK9, the LDL/LDLR complex undergoes endocytosis. In the acidic endosome, the LDLR dissociates from LDL and is recycled back to the hepatocyte surface to receive another LDL cargo, while the LDL undergoes lysosomal degradation. When LDL binds to LDLR in the presence of PCSK9, the LDLR does not dissociate from LDL and is instead channeled toward lysosomal degradation. This action reduces LDLR density on the surface of hepatocytes, allowing levels of LDL-C to rise.

**Figure 2:**
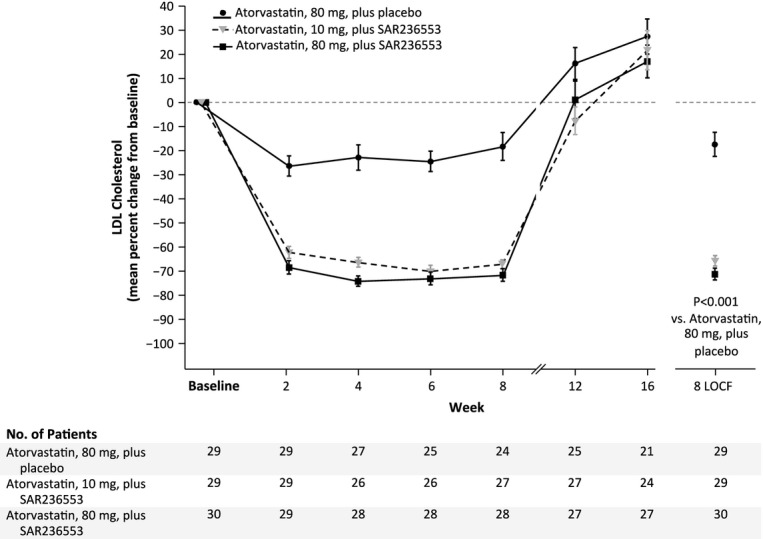
Effect of PCSK9 inhibition on low-density lipoprotein (LDL) cholesterol levels Patients with primary hypercholesterolemia were treated with atorvastatin 10 mg during a run-in period and then randomly assigned to treatment with atorvastatin 80 mg daily with alirocumab (SAR236553) every 2 weeks, atorvastatin 10 mg daily with alirocumab (SAR236553) every 2 weeks, or atorvastatin 80 mg daily with placebo every 2 weeks. Alirocumab produced a profound reduction in LDL-C compared with placebo. There was minimal additional LDL-C reduction when alirocumab was administered with atorvastatin 80 mg, compared with atorvastatin 10 mg daily [Reproduced with permission from Roth*et al* (2012)].

Gain-of-function mutations in PCSK9 are a cause of familial hypercholesterolemia and premature coronary heart disease, while loss-of-function mutations are associated with lifelong low levels of LDL-C and substantially reduced coronary heart disease risk (Davignon*et al*, 2010). Statins increase the expression of PCSK9, an effect that may attenuate the LDL-C lowering produced with these agents (Dubuc*et al*, 2004). Conversely, therapeutic interference with PCSK9 expression or action with small interfering RNA or monoclonal antibody (Frank-Kamenetsky*et al*, 2008; Dias*et al*, 2012; Stein*et al*, 2012) allows greater recycling of LDLR to the hepatocyte surface, resulting in lower LDL-C levels. Phase 2 clinical trials have demonstrated efficacy of PCSK9 antibody in achieving LDL-C reductions of up to 73% when added on to statin background therapy. In this trial when atorvastatin dose was increased from 10 to 80 mg daily and PCSK9 antibody was added, LDL-C was reduced by 73%, as compared to a 17% reduction with atorvastatin 80 mg alone (Roth*et al*, 2012) (Fig2). Large Phase 3 clinical trials are testing the efficacy of PCSK9 antibodies to reduce major adverse cardiovascular events (NCT01764633 and NCT01663402). Because inhibition of PCSK9 does not impair hepatic lipid export, this approach is unlikely to cause hepatic steatosis. However, it is premature to assess whether very low LDL-C levels achieved in some patients treated with PCSK9 antibodies will lead to adverse long-term effects, or whether immunologic reactions to the antibodies will occur.

### Lipoprotein (a)

**Figure 3:**
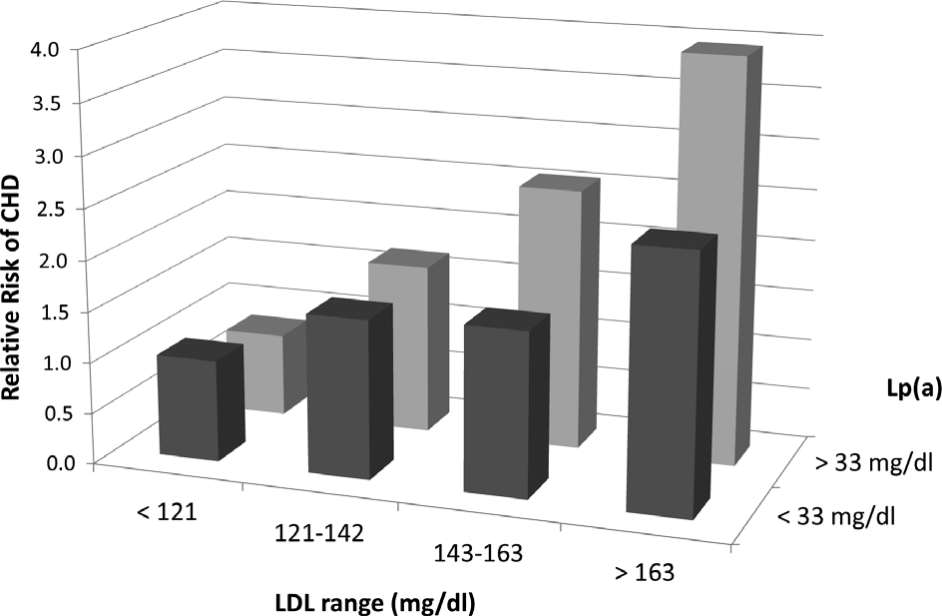
Relation of Lp(a) and LDL-C to CHD risk Prospective cohort study of 9,133 middle-aged men, without history of CHD and not on lipid-lowering therapy. In models adjusted for smoking, diabetes, and hypertension, levels of Lp(a) and LDL-C were independently predictive of incident CHD [Drawn from data in Luc*et al* (2002)].

Lipoprotein (Lp)(a) is an LDL-like particle in which apoB is covalently bound to apo(a). Both moieties may mediate atherogenicity. Apo(a) is structurally similar to plasminogen and can interfere with plasminogen activation and fibrinolysis (Hancock*et al*, 2003). Lp(a) can also carry oxidized phospholipids that may be pro-inflammatory (Wiesner*et al*, 2013). Epidemiologic data suggest that a high level of Lp(a) is an independent risk factor for the development of cardiovascular disease (Erqou*et al*, 2009). Serum levels of Lp(a) are influenced by genetic factors (Boerwinkle*et al*, 1992), but not by diet or lifestyle factors (Thomas*et al*, 1997). In cohort studies, cardiovascular risk associated with Lp(a) is independent of smoking, diabetes, hypertension, as well as LDL-C (Luc*et al*, 2002) (Fig3).

Niacin exerts the greatest Lp(a) lowering effects among currently available lipid-modifying therapies. However, a 21% average reduction in Lp(a) with niacin was not associated with a lower event rate in the niacin arm, compared with placebo. This may indicate either that Lp(a) is not causally related to risk or that niacin produced other undesirable effects that negated a benefit of raising Lp(a) (Boden*et al*, 2011). PCSK9 antibodies lower Lp(a) concentrations by mechanisms as yet unknown (McKenney*et al*, 2012). Lipoprotein apheresis can also remove Lp(a) from the circulation. In a prospective observational study in patients with elevated Lp(a), the initiation of lipoprotein apheresis was accompanied by a reduced incidence of cardiovascular events (Leebmann*et al*, 2013). Novel approaches to the reduction of Lp(a) involve the use of antisense oligonucleotides. Mipomersen reduces Lp(a) by approximately 25% (Visser*et al*, 2012) and antisense oligonucleotide to apo (a) has been shown to lower Lp(a) concentrations in a Phase 1 clinical study (Viney*et al*, 2013). The latter approach may provide the best tool to determine whether Lp(a) plays a direct, causative role in cardiovascular disease.

## Targets for reduction of triglyceride-rich lipoproteins

Triglyceride-rich lipoproteins include VLDL, chylomicrons and the remnant particles formed from them upon the action of lipases. These particles contain apoB, C, and E and are believed to be atherogenic.*Post hoc* and meta-analyses of statin trials suggest that higher level of triglyceride-rich lipoproteins and their associated cholesterol correlate with higher cardiovascular risk (Miller*et al*, 2008; Bruckert*et al*, 2011).

Niacin and fibrates reduce triglycerides and have been in therapeutic use for many years. Early studies of these agents indicated a cardiovascular benefit. Although subsequent studies utilizing these agents in addition to statins failed to show overall benefit (Boden*et al*, 2011),*post-hoc* analyses suggest that patients with significant baseline hypertriglyceridemia benefit from fibrates (Lee*et al*, 2011). Other strategies to reduce triglyceride-rich lipoproteins, outlined below, are under active investigation.

### Omega-3 fatty acids

Population studies demonstrate that cohorts with low levels of eicosapentaenoic acid (EPA) and docosahexaenoic acid (DHA) have higher levels of cardiovascular risk. Omega-3 fatty acids are found in fish oils and plants and have been observed to lower triglyceride levels, improve endothelial function, and have favorable effects on thrombotic and arrhythmic potential. The triglyceride-lowering effects of omega-3 fatty acids are due to multiple mechanisms, including stimulation of transcription factors such as PPAR α, increased hepatic and extrahepatic fatty acid oxidation, and decreased hepatic incorporation of fatty acids into triglyceride resulting in decreased VLDL synthesis (Shearer*et al*, 2012). Despite efficacy in triglyceride lowering, data indicating a cardiovascular benefit alone or when added to statins are inconclusive (Rizos*et al*, 2012). A concentrated formulation of EPA, eicosapent ethyl, is being evaluated for effects on lipoproteins, biomarkers of cardiovascular risk, and clinical cardiovascular outcomes (Ballantyne*et al*, 2012; NCT01492361).

### Apolipoprotein C-III

**Figure 4:**
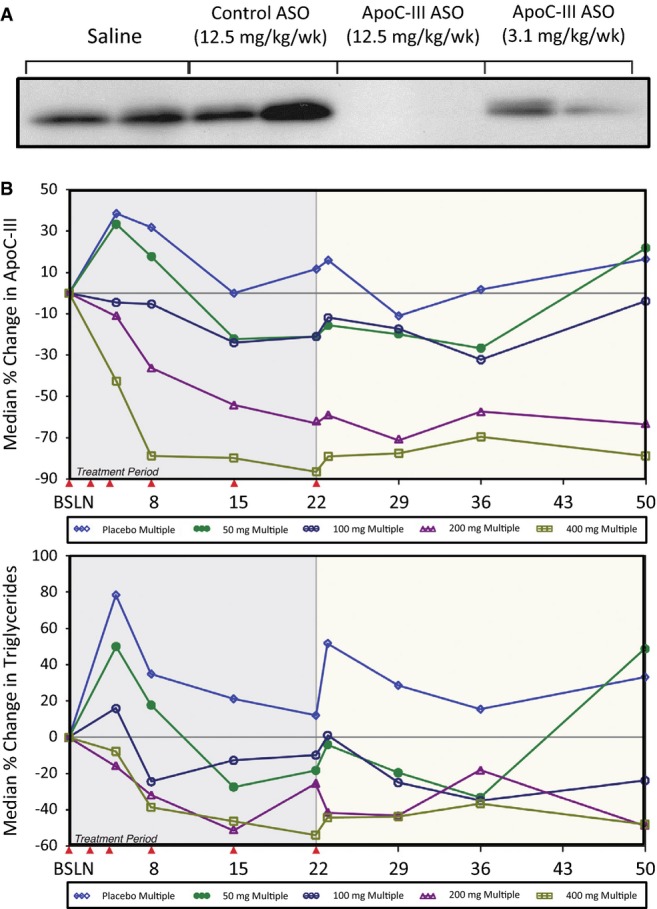
Effects of apolipoprotein C-III antisense oligonucleotide (ASO) (A) Effects of VLDL-associated apoC-III in mice. Mice were administered control ASO or one of two doses of apoC-III ASO for 6 weeks. Western blot demonstrates a dose-dependent reduction of VLDL-associated apoC-III protein. (B) Effects of apoC-III ASO on circulating apoC-III and triglycerides in healthy human volunteers. ASO was administered by subcutaneous injections with loading dose followed by three weekly doses of either 50, 100, 200, or 400 mg. A dose-dependent response was demonstrated, with up to 75% reduction in apoC-III and 50% reduction in triglycerides, sustained over 50 days [Reproduced with permission from Graham*et al* (2013)].

ApoC-III is located on the surface of triglyceride-rich lipoproteins including VLDL, chylomicrons, and their remnants and is implicated in cardiovascular disease. High levels of apoC-III are associated with delayed clearance of VLDL (Aalto-Setala*et al*, 1992) and may exert pro-inflammatory effects at the level of the artery wall (Kawakami*et al*, 2006). Further supporting its atherogenic role, those with dysfunctional apoC-III have lower cardiovascular risk. In two recent genetic studies, subjects with loss-of-function mutations in the gene encoding apoC-III were noted to have 39–44% lower levels of triglycerides and ˜40% lower risk of coronary heart disease (Jørgensen*et al*, 2014; TG and HDL Working Group, 2014). An apoC-III antisense oligonucleotide has demonstrated substantial reductions in apoC-III, triglycerides (Fig4) in addition to elevations of HDL-C, without inducing hepatic steatosis (Graham*et al*, 2013). This agent is currently being evaluated in patients with severe hypertriglyceridemia. The impact of this approach on cardiovascular risk has not yet been studied. ApoC-III is also present on HDL particles. It is uncertain whether ApoC-III on HDL conveys pro- or anti-atherogenic properties (Cho, 2009; Riwanto*et al*, 2013).

**Figure 5:**
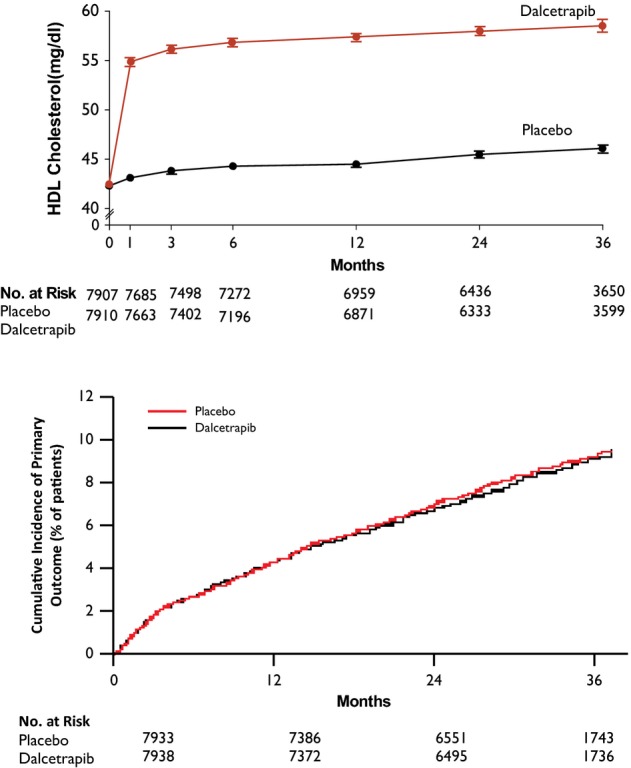
Effect of dalcetrapib on HDL-C and cardiovascular risk after ACS Dalcetrapib was administered to patients with recent ACS on background statin therapy. While dalcetrapib raised HDL-C by 30% compared with placebo (top), there was no difference between groups in the primary endpoint of death from coronary heart disease, non-fatal myocardial infarction, hospitalization for unstable angina, resuscitation after cardiac arrest, or stroke from presumed atherothrombotic cause (bottom) [Reproduced with permission from Schwartz*et al* (2012)].

### Diacylglycerol acyltransferase (DGAT)

DGAT is an enzyme expressed in small intestine, liver, and adipose tissue. DGAT isozyme 1 is involved in a final committed step of triglyceride synthesis from diacylglycerol. Inhibition of the enzyme may work to reduce serum triglyceride concentrations. Small molecule inhibitors have been developed, but clinical application is likely to be limited by gastrointestinal side effects (Denison*et al*, 2014).

### Apolipoprotein E

ApoE is a ligand for receptor-mediated clearance of chylomicron and VLDL remnants, which are particles that may promote atherosclerosis directly or through the action of lipases to release toxic products of lipolysis (Goldberg*et al*, 2011). ApoE also participates in the biogenesis of HDL, as it is recycled from triglyceride-rich lipoproteins in the liver (Zannis*et al*, 2008). ApoE mimetic peptides have been developed that exert anti-inflammatory effects, promote HDL function*in vitro* (Zhao*et al*, 2011), and oppose atherosclerosis*in vivo*. In LDL receptor knockout mice prone to atherosclerosis, ApoE mimetic peptides reduce plasma cholesterol and the extent of vascular lesions (Handattu*et al*, 2013). One ApoE mimetic peptide, AEM-28, has been granted orphan drug status by the US Food and Drug Administration and may enter early phase clinical testing.

## HDL and related atheroprotective lipoproteins

HDL particles and associated apolipoprotein A-I are believed to be anti-inflammatory and atheroprotective (Besler*et al*, 2012). Experimental data support the concept that HDL and/or apoA-1 have vascular anti-inflammatory effects. For example, administration of reconstituted HDL to animals has been shown to reduce expression of adhesion molecules in vascular endothelium and to attenuate the inflammatory response to experimental arterial injury (Nicholls*et al*, 2005). Additionally, HDL may promote the expression of endothelial nitric oxide synthase and exert anti-thrombotic effects (Barter*et al*, 2004). Several large population studies reported an inverse relationship between HDL-C levels and prospective risk of cardiovascular events (Castelli*et al*, 1986), independent of atherogenic lipoprotein levels (Di*et al*, 2009).

HDL facilitates reverse cholesterol transport and cholesterol efflux from peripheral tissues. Subpopulations of HDL interact with different membrane bound transporters such as ATP binding cassette A1 (ABCA1), ATP binding cassette G1 (ABCG1), and scavenger receptor-BI (SR-BI) to assist in cholesterol efflux (Acton*et al*, 1996; Vaughan & Oram, 2006). Cholesterol is transferred to HDL particles, esterified by lecithin/cholesterol acyltransferase (LCAT), and then transported to the liver for excretion.

Yet, agents that substantially raised HDL-C failed to demonstrate corresponding clinical benefit (Boden*et al*, 2011; Schwartz*et al*, 2012). In epidemiologic analyses, cardiovascular risk is mainly evident at the lowest levels of HDL-C and does not necessarily support risk reductions by raising HDL-C to very high levels. Moreover, the protein cargo of HDL may be altered in patients with vascular disease and less protective (Besler*et al*, 2012; Riwanto*et al*, 2013). Despite these caveats, numerous approaches to modify HDL concentration and/or function continue to be evaluated.

### Niacin

Of currently available drugs, niacin is the most effective at raising HDL-C, increasing levels by as much as 33% (Illingworth*et al*, 1994). The lipid-modifying effects of niacin are thought to be mediated by activation of G protein-coupled receptors in adipose tissue and liver leading to reduced lipolysis and hepatic VLDL synthesis, respectively (Kamanna & Kashyap, 2008). While early data demonstrated cardiovascular benefit (Canner*et al*, 1986), more recent clinical trials have failed to demonstrate a clinical benefit of niacin in statin-treated patients (Boden*et al*, 2011). These data suggest that alternative HDL-targeted therapies are required if this approach is to achieve reductions in cardiovascular risk.

### Cholesteryl ester transfer protein inhibitors

Cholesteryl ester transfer protein (CETP) promotes the movement of esterified cholesterol from HDL to VLDL and LDL particles, in exchange for triglyceride. Lower CETP activity has been associated with higher HDL-C levels and lower cardiovascular event rates in some, but not all analyses (Thompson*et al*, 2008; Ritsch*et al*, 2010). Clinical development of torcetrapib was halted prematurely due to increased cardiovascular morbidity and mortality (Barter*et al*, 2007). This was potentially attributed to off-target effects including upregulation of cortisol and aldosterone synthesis and elevated blood pressure (Forrest*et al*, 2008).

Dalcetrapib is a CETP inhibitor without effects on neurohormones and with minimal if any effect on blood pressure. However, despite increasing HDL-C by approximately 30%, dalcetrapib had no effect on vascular endothelial function (Luscher*et al*, 2012), carotid atherosclerosis (Fayad*et al*, 2011), or cardiovascular events (Schwartz*et al*, 2012) (Fig3). Notably, dalcetrapib has minimal effects on LDL-C. Anacetrapib and evacetrapib are CETP inhibitors with pronounced LDL-lowering as well as HDL-raising effects (Nicholls*et al*, 2011a,b; Gotto*et al*, 2014) that are undergoing evaluation in Phase 3 cardiovascular outcome trials.

### Regulators of apolipoprotein A-I synthesis

Hepatic production of apoA-I results in generation of nascent, lipid-deplete HDL particles, which enter the systemic circulation and carry out their biological activities. The bromodomain and extra-terminal (BET) domain inhibitor, RVX-208, induces hepatic apoA-I synthesis. RVX-208 administration increased cholesterol efflux capacity in non-human primates (Bailey*et al*, 2010). In statin-treated patients with coronary disease, RVX-208 produced modest dose related increases in apoA-I and HDL-C. This was driven predominantly by increases in large HDL particles, suggesting that cholesterol mobilization to functional HDL particles was occurring (Nicholls*et al*, 2011a,b). In a subsequent study in patients with low HDL-C levels, modest coronary plaque regression was demonstrated on serial intravascular ultrasound, but changes with RVX-208 did not differ significantly from placebo (Nicholls*et al*, 2013). Increases in hepatic transaminase levels were more frequent with RVX-208 than placebo. The effect of RVX-208 on cardiovascular outcomes remains unknown.

### HDL infusion therapy

Infusing HDL or apoA-1 presents a conceptually simple, but challenging approach. Infusion of lipid-deplete forms of HDL has favorable effects on atherosclerotic plaque, endothelial function, and surrogate markers of reverse cholesterol transport (Spieker*et al*, 2002; Tardy*et al*, 2014). Intravenous infusions of complexes containing the apoA-I variant, apoA-I Milano and phospholipid (ETC-216) resulted in regression of coronary atherosclerosis measured by serial intravascular ultrasound in patients with a recent acute coronary syndrome (Nissen*et al*, 2003). Another approach has been infusion of complexes of wild-type apoA-I and phospholipid (CSL-111). This agent produced a trend toward regression of coronary atherosclerotic plaque on serial intravascular ultrasound imaging, but liver transaminase elevations required cessation of testing of the highest dose (Tardif*et al*, 2007). Challenges in producing large quantities of infusible HDL-mimetic complexes appear to have been overcome, and development of several agents is proceeding.

### Delipidated HDL

A unique approach to HDL therapeutics involves the selective delipidation of a patient's HDL, which is subsequently reinfused. Potentially, the lipid-poor HDL has greater capacity for cholesterol efflux. A small imaging study demonstrated coronary plaque regression with this approach (Waksman*et al*, 2010).

### Mimetic peptides

Synthetic production of apoA-I presents a considerable challenge. In contrast, an alternative approach is in preparing short peptide sequences that lack genetic homology to apoA-I, but similarly form an amphipathic helix and associate effectively with lipids. Preclinical studies using these peptides have demonstrated favorable effects on cholesterol efflux, LCAT activation, inflammatory and oxidative pathways, and ultimately atherosclerotic plaque (Datta*et al*, 2001; Bielicki*et al*, 2010). CER-001, an HDL-mimetic made up of apoA-I and phospholipids, has been associated with reduction of vascular inflammation and atherosclerotic regression after short-term administration in mice (Tardy*et al*, 2014) as well as cholesterol mobilization in humans. In a Phase 2 trial, the primary endpoint of reducing atheroma volume compared with placebo was not reached (Tardif*et al*, 2014). However, the safety profile has thus far been acceptable, and the drug may be effective in reverse lipid transport. Thus, further study in clinical trials may be warranted.

### Reverse cholesterol transport targets

Increasing expression of ABCA-1 provides a potential opportunity to target a major factor implicated in cholesterol efflux, as opposed to simply increasing carrier (apoA-1 or HDL) capacity. However, work in this field has not advanced clinically.

Similarly, development of chemical inhibitors of microRNA elements implicated in the regulation of lipid metabolism may be beneficial. MicroRNA (miR)-33 is an intronic microRNA that suppresses ABCA-1 expression and reduces HDL-C levels (Rayner*et al*, 2010). Early preclinical experience with miR-33 inhibitors demonstrates variable effects on atherosclerosis in animal models (Marquart*et al*, 2013; Rotllan*et al*, 2013). Given the central role of LCAT in reverse cholesterol transport by HDL, LCAT agonists have been developed and are undergoing preclinical evaluation (Chen*et al*, 2012).

### HDL functional modification

Given disappointing results of HDL-C raising therapies in recent clinical trials (Barter*et al*, 2007; Boden*et al*, 2011), there is increasing interest in qualitative features of HDL function. HDL particles circulate as a heterogeneous population of lipoproteins, differing in size, shape, protein, and lipid composition (Rosenson*et al*, 2011). Furthermore, mass spectrometry has demonstrated more than 100 individual proteins that can be carried on HDL particles, many with activities beyond lipid metabolism, and many that are altered in patients with vascular disease compared with healthy control (Riwanto*et al*, 2013). Data indicating that cholesterol efflux capacity is a better predictor of atherosclerotic burden than HDL-C concentration suggest that HDL quality may be a better indicator of the efficacy of novel HDL-targeted therapies (Hafiane*et al*, 2014).

## Other approaches affecting lipoprotein metabolism

### Nuclear receptors

Nuclear receptors including liver X receptors (LXRs), peroxisome proliferator-activated receptors (PPARs), and farnesoid X receptors (FXR) play a central role in lipid metabolism. The reader is referred to other reviews for discussion of bile acid receptors, including FXRs (Porez*et al*, 2012).

### Liver X receptor

LXRs are thought to serve as cholesterol sensors that when activated, increase biliary cholesterol excretion, reduce intestinal cholesterol absorption, and promote reverse cholesterol transport. LXR agonists have been demonstrated to attenuate atherosclerosis in animal models (van der Hoorn*et al*, 2011). However, a potentially limiting factor in clinical treatment with LXR agonists is the stimulation of hepatic lipogenesis (Fievet & Staels, 2009). LXR agonists have been demonstrated to attenuate atherosclerosis in animal models (Kappus*et al*, 2014). Some LXR agonists have been evaluated in early stage clinical trials (Katz*et al*, 2009; NCT00796575, NCT00836602, NCT01651273, NCT01651273), but their development for cardiovascular disease appears to have been halted. It remains uncertain whether agents can be developed to exploit the potential benefits of LXR activation while avoiding hypertriglyceridemia and hepatic steatosis.

### Peroxisome proliferator-activated receptors (PPARs)

PPARs (α, γ, or δ) play important roles in the regulation of fatty acid and lipoprotein metabolism. Among their principal actions, PPARα promotes fatty acid oxidation in liver and muscle, lowers circulating triglycerides and apoC-III, and raises HDL-C, PPARγ promotes fatty acid uptake by adipocytes and lowers circulating fatty acids, and PPARδ promotes fatty acid oxidation in muscle and adipose tissue. Fibrate and thiazolidinedione drugs are ligands of PPARα and PPARγ, respectively. Early studies showed that gemfibrozil, a fibrate, reduced cardiovascular morbidity and mortality (Frick*et al*, 1987; Robins*et al*, 2001). However, subsequent studies evaluating the addition of fibrates to statins have not demonstrated clinical benefit (ACCORD Study Group, 2010). Yet, meta-analysis indicates that fibrates may confer clinical benefit in patients with triglyceride levels at least 200 mg/dl, even with statin co-treatment (Bruckert*et al*, 2011). In this regard, it may be premature to conclude that fibrates are ineffective in reducing residual cardiovascular risk. Composite evidence suggests cardiovascular efficacy of pioglitazone, a thiazolidinedione (Lincoff*et al*, 2007). Yet, a dual PPAR-α/γ activator has failed to demonstrate clinical efficacy (Lincoff*et al*, 2014). Selective PPAR-δ (Choi*et al*, 2012) and dual PPAR-α/δ activators (Cariou*et al*, 2013) have been evaluated in early phase clinical trials, but their cardiovascular efficacy remains untested.

### Phospholipase inhibitors

The phospholipase A2 family of enzymes hydrolyzes the sn-2 ester bond of phospholipids in cell membranes and circulating lipoproteins, generating metabolites that may influence vascular function and inflammation. Secretory phospholipase A2 (sPLA2) activity results in the generation of smaller, more atherogenic LDL particles and the generation of pro-inflammatory and oxidative metabolites within the artery wall (Hurt-Camejo*et al*, 2001). However, a Phase 3 study evaluating the sPLA2 inhibitor varespladib in patients with acute coronary syndrome demonstrated increased risk of recurrent myocardial infarction (Nicholls*et al*, 2014). Lipoprotein-associated phospholipase A2 (Lp-PLA2) is largely associated with LDL. Elevated plasma concentrations or activity is associated with greater cardiovascular risk. In early phase studies, the Lp-PLA2 inhibitor, darapladib, demonstrated favorable effects on lipid and inflammatory biomarkers and a reduction of the volume of necrotic core within atherosclerotic plaques (Serruys*et al*, 2008). However, a large outcome trial failed to demonstrate a benefit of darapladib on cardiovascular death, myocardial infarction, or stroke (STABILITY Investigators, 2014).

## Conclusion

LDL-C, triglyceride-rich lipoproteins, and HDL-C may each play a role in the development and progression of atherosclerosis and its complications. For more than a quarter century, statins have been the central element of lipid-modifying therapy to reduce cardiovascular risk. However, residual cardiovascular risk on statin treatment remains high. To date, no agent added to statins has yet been proven to provide incremental clinical benefit. However, novel approaches to further reduce LDL, to target triglyceride-rich lipoproteins, and to increase the concentration or functionality of HDL are under evaluation in basic investigations and in clinical trials, holding promise that tools to further reduce cardiovascular morbidity and mortality may be forthcoming.

**Pending issues**

Develop novel lipid-modifying therapies, utilizing small molecule, monoclonal antibody, antisense oligonucleotide, and small interfering RNA approaches

Test the hypothesis that lowering LDL and/or other apoB-containing lipoproteins below levels achieved with statins provides incremental clinical benefit to patients

Test the hypothesis that agents that increase the concentration or enhance the function of HDL reduce cardiovascular risk when added to statins

## Conflict of interest

Dr. Rose Do, through her institution, has received research support from Sanofi. Dr. Stephen Nicholls has received research support from Amgen, AstraZeneca, Cerenis, Novartis, Resverlogix, Eli Lilly, LipoScience, and Roche. He also serves as a consultant for Amgen, AstraZeneca, Atheronova, Boehringer Ingelheim, Cerenis, CSL Behring, Eli Lilly, LipoScience, Merck, Novartis, Pfizer, Resverlogix, Roche, and Takeda. Dr. Gregory Schwartz, through his institution, has received research grants from Sanofi, F. Hoffmann-La Roche, and Anthera Pharmaceuticals.

## For more information

International Atherosclerosis Society – scientific organization dedicated to advance research and therapy in the area of atherosclerosis and its complications. http://athero.org

National Lipid Association – scientific and medical organization dedicated to advance lipid management in clinical medicine. https://www.lipid.org/

National Heart Lung and Blood Institute of the National Institutes of Health – US government agency dedicated to basic and clinical research in cardiovascular disease, including atherosclerosis. Web site provides information for patients and healthcare/scientific professionals. http://www.nhlbi.nih.gov/health/health-topics/topics/atherosclerosis/

## Abbreviations

Apheresis: a procedure used to remove a substance from blood circulation. Whole blood is drawn from the body by large catheter and separated into different blood components. The desired substance can then be removed, with reinfusion of remaining blood products to the body.
Atherosclerosis: the process of cholesterol plaque deposition and inflammation along arterial walls. It can result in obstruction to blood flow and a tendency to thrombosis with sudden vessel occlusion.
Chylomicrons: large lipoprotein particle that carries dietary lipid and is rich in triglyceride.
Foam cells: macrophages or immune cells containing excess cholesterol, largely due to uptake of oxidized LDL.
High-density lipoprotein (HDL): ApoA1-containing lipoprotein; major function is reverse transport from peripheral sites to liver for excretion.
Intermediate density lipoprotein: formed by action of lipases on very low-density lipoproteins, resulting in increased cholesterol and decreased triglyceride content and smaller size, compared with VLDL.
Lipoprotein: particles consisting of surface proteins and phospholipid and a neutral lipid core, functioning to transport of lipids throughout the circulation.
Low-density lipoprotein (LDL): ApoB-containing lipoprotein. Major carrier of cholesterol esters to peripheral cells.
Myopathy: muscle disease that in general describes muscle weakness or pain.
Hepatic steatosis: caused by an imbalance of lipid uptake or synthesis compared with clearance from the liver.
Very low-density lipoprotein: carries triglycerides and cholesterol from liver to peripheral tissue and contains apolipoproteins B, C, and E.
